# Attentional switching between perception and memory: Examining asymmetrical switch costs

**DOI:** 10.3758/s13414-023-02665-w

**Published:** 2023-02-28

**Authors:** Caro Hautekiet, Sam Verschooren, Naomi Langerock, Evie Vergauwe

**Affiliations:** 1 Faculty of Psychology and Educational Sciences, University of Geneva, 40 bd du Pont d’Arve, 1211 Genève 4, Switzerland; 2 Center for Cognitive Neuroscience, Duke University, Durham, NC USA; 3 Department of Experimental Psychology, Ghent University, Ghent, Belgium

**Keywords:** Attention, Working memory, Focus of attention, Asymmetrical switch costs, Switching

## Abstract

Attention can be defined as a mechanism for the selection and prioritization of elements among many. When attention is directed to a specific piece of information, this information is assumed to be in the focus of attention. On a day-to-day basis, we need to rely on efficient switching between information we are holding in working memory (internal modality) and information presented in the world around us (external modality). A recent set of studies investigated between-modality attentional switches and found that there is an asymmetrical switch cost for switching between the internal and external focus of attention (Verschooren et al., 2020, *Journal of Experimental Psychology: Human Perception and Performance, 46*[9], 912–925; Verschooren, Liefooghe, et al., 2019a, *Journal of Experimental Psychology: Human Perception and Performance, 45*[10], 1399–1414). In particular, participants switched on a trial-by-trial basis between an internal task using stimuli retrieved from memory and an external task using on-screen presented stimuli. A larger cost was found when switching from the external modality towards the internal modality than the other way around. The authors found that this cost asymmetry could be best explained in terms of associative interference (i.e., differences in shielding efficiency against the memory traces from the competing task set). The present study aimed to replicate the asymmetrical switch cost (Experiment 1) and investigate whether an alternative explanation in terms of stimulus strength can account for the asymmetrical switch cost (Experiment 2). Overall, the results confirm the presence of a subtle, asymmetrical switch cost, but we observed little to no contribution of stimulus strength.

Working memory is a limited-capacity maintenance system responsible for processing and keeping information available over a short period (Baddeley & Hitch, 1974). Perception, on the other hand, can be understood as the comprehension and processing of sensory information (i.e., in the world around us). In both modalities, it is possible to focus on specific information through attention, which can be defined as a mechanism for the selection and prioritization of elements among many (e.g., Chun et al., 2011; Desimone & Duncan, 1995). In working memory, this is done via internal attention; in perception, via external attention (e.g., Chun et al., 2011). When attention is concentrated on a specific item in working memory, this item is assumed to be in the internal focus of attention (e.g., Garavan, 1998; McElree, 2006; Oberauer, 2002, 2009). Similarly, when attention is concentrated on a specific item in the world around us, this item is assumed to be in the external focus of attention (e.g., Duncan, 1980; Eriksen & St James, 1986; Treisman & Gormican, 1988). Overall, there seems to be an agreement that internal and external attention are closely linked and share some properties (e.g., Awh et al., 1998; Kiyonaga & Egner, 2013, 2014a, 2014b; Olivers, 2008; see Oberauer, 2019, for a recent review).

Over the past years, the relationship between these two foci of attention has been investigated in at least two different ways. One approach has been to examine whether effects typically found in external attention can also be found in internal attention (e.g., a working memory Stroop effect, Kiyonaga & Egner, 2014a). Another approach, and this will be the main focus of the current study, examined switching between the internal and external focus of attention.

## Asymmetrical switch costs between the internal and external focus of attention

Currently, little is known about how flexibly we can switch between the internal and external focus of attention (see Verschooren, Schindler, et al., 2019b, for a review). A recent set of studies investigated attentional switching between an item held in the internal focus of attention and an item in the external focus of attention, and vice versa (Verschooren et al., 2020; Verschooren, Liefooghe, et al., 2019a). In these studies, participants switched on a trial-by-trial basis between an internal task using stimuli retrieved from memory and an external task using stimuli presented on-screen. In the internal task, participants were instructed to compare an on-screen presented target to an in-memory kept item, previously presented in the indicated location (i.e., memorized beforehand). In the external task, participants were instructed to compare an on-screen presented target to an on-screen item presented in the indicated location. In half of the trials, the same modality was repeated, meaning that there were two consecutive internal trials (i.e., internal repeat) or external trials (i.e., external repeat). In the other trials, the modality was switched, meaning that an external trial was followed by an internal trial (i.e., internal switch) or an internal trial was followed by an external trial (i.e., external switch). The authors found an asymmetrical cost for switching between the internal and external focus of attention; participants were slower to switch from an item held in the external focus of attention to an item in the internal focus of attention than vice versa (Verschooren et al., 2020; Verschooren, Liefooghe, et al., 2019a).

Verschooren et al. (2020) adjudicated between different theoretical accounts for this asymmetry and found that an associative interference account explained it best. This account originates from the task switching literature and entails that differences in shielding efficiency can explain the asymmetrical switch cost (Mayr et al., 2014). In the task switching literature, when switching between two tasks with a different difficulty (e.g., color naming and word naming in a Stroop task; Allport et al., 1994), costs are typically higher for switching to the easier, dominant task (i.e., word naming) compared with switching to the more difficult, nondominant task (i.e., color naming; see Kiesel et al., 2010 for a review). In particular, during repeat trials, the dominant task can be efficiently shielded against the memory traces from the nondominant task. This is not the case for the nondominant task, which suffers a constant flow of interference from the dominant task during repeat trials. In both cases, when a switch occurs, working memory needs to update, making it more vulnerable to intrusion from the other task (see also Kessler, 2017). On switch trials, the dominant task receives a sudden burst of interference coming from the nondominant task. The nondominant task already receives a constant stream of interference from the dominant task on repeat trials, and thus is barely affected by the interference on switch trials. Together, this results in a larger difference between switch and repeat trials for the dominant task, while there is only a small difference in performance for the nondominant task.

Given the larger cost for switching from the external to the internal task, the only way the associative interference account fits the data of Verschooren et al. (2020) is if one assumes that the external task is the nondominant task, whereas the internal task is the dominant task. This implies that comparing an on-screen item to an in-memory item (i.e., internal task) is the easier (dominant) task while comparing two on-screen items (i.e., external task) is the more difficult (nondominant) task. This assumption seems rather counterintuitive (but see Verschooren & Egner, 2022). Additionally, the associative interference account is based on processes in procedural working memory^1^ (i.e., on the task set level). However, we believe there might be at least one specific alternative account that is based on processes in declarative working memory (i.e., on the representational, item level). Therefore, we decided to attempt to replicate the asymmetrical switch cost as well as test one specific alternative account.

In particular, we investigated whether the asymmetrical switch cost can be explained by an asymmetry in stimulus strength between the internal and external items. While internal items are mental representations, external items are present in the environment. Therefore, one could assume that internal, in-memory items have a weaker representation than external, on-screen items. When a switch is made from external to internal, attention needs to be refocused from stronger, on-screen items to weaker, in-memory representations.^2^ In comparison, when a switch is made from internal to external, attention needs to be refocused from weaker, in-memory representations to stronger, on-screen items. In the former, we would expect a certain cost to switch attention, while this is not, or less, the case in the other direction. Taken together, differences in stimulus strength could potentially explain the observed asymmetrical switch cost.

Therefore, in Experiment 1, we aimed to replicate the findings by Verschooren et al. (2020, Experiment 1). If there is indeed an asymmetrical switch cost, we expected to find an interaction between the task modality (internal vs. external) and trial type (repeat vs. switch). Such that, in both modalities, reaction times (RTs) for the switch trials would be larger than for repeat trials and that the difference between repeat and switch trials (i.e., switch cost) would be substantially larger in one of the two modalities. Specifically, here, we expected that the internal switch cost would be substantially larger than the external switch cost. It is important to note that by aiming to replicate the asymmetrical switch cost as observed by Verschooren et al. (2020), we did not aim to test the associative interference account. In Experiment 2, we investigated how the asymmetry is affected by reducing the difference in stimulus strength between internal and external items, by degrading the external items. If this drastically reduces the asymmetry, this would indicate that differences in stimulus strength play an important role in the previously found asymmetrical switch cost.

## Methods

Both experiments were preregistered prior to conducting the studies on the Open Science Framework (OSF), see https://osf.io/75t4m for Experiment 1 and https://osf.io/ud96e for Experiment 2. There were no deviations from the preregistrations. The two experiments were conducted with significant overlap in time and will be reported together.

### Participants

Following our preregistration, the number of participants was determined using Bayesian sequential hypothesis testing. Specifically, we planned to start with 40 participants and to continue to increase by five participants (with max. 60) until we obtained a Bayes factor (BF) of 10 for or against the presence of the interaction of interest in each experiment, after applying the preregistered exclusion criteria (see BANOVA with two factors described in Data Analysis and Results). In total, 60 participants (53 females, seven males, mean age = 20.46 years) in Experiment 1 and 60 participants (48 females, 12 males, mean age = 22.45 years) in Experiment 2 from the University of Geneva took part in exchange for course credits. All participants signed an informed consent before participating. The ethical commission board at the University of Geneva approved both experiments.

### Materials

**Fig. 1:**
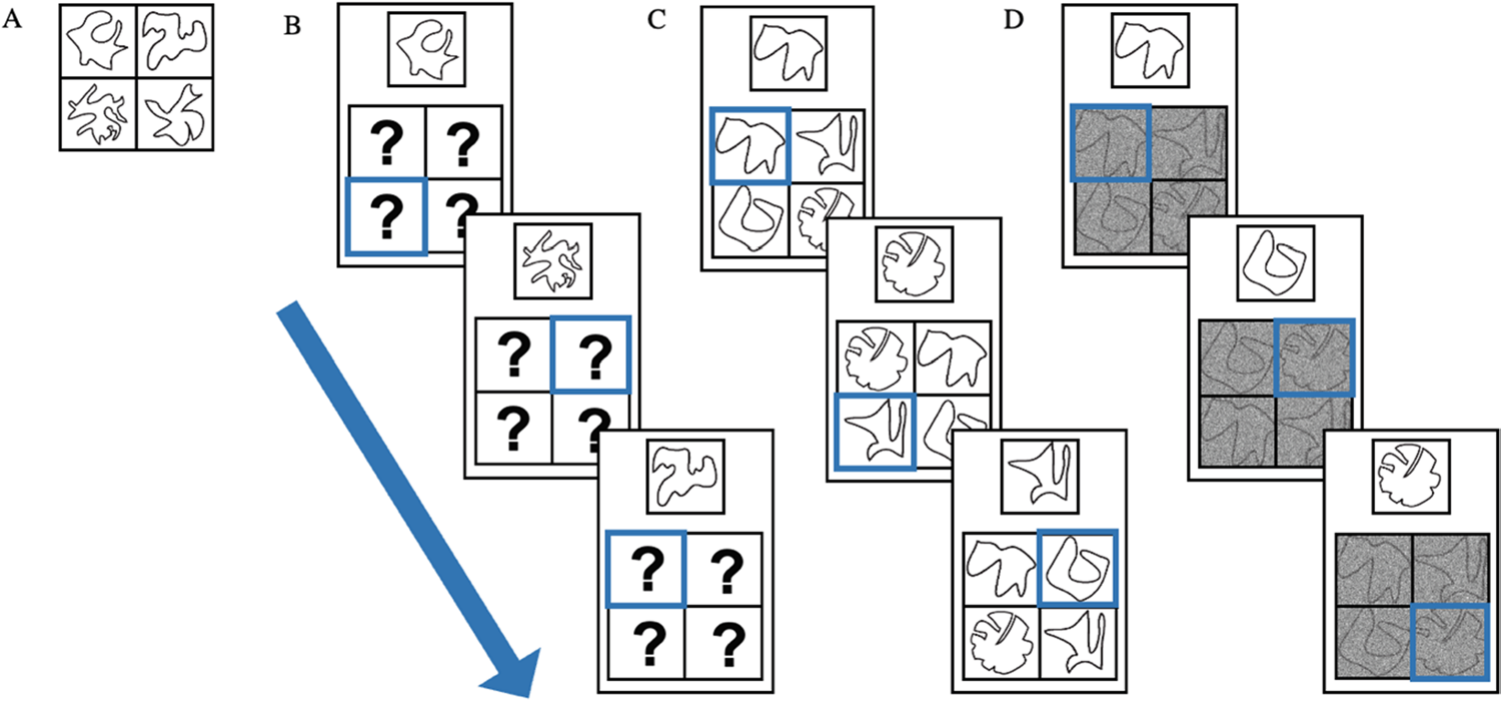
An example of (**A**) the to-be-memorized internal stimuli from Experiments 1 and 2, (**B**) trials of the internal familiarization task from Experiments 1 and 2, (**C**) trials of the external familiarization task from Experiment 1, and (**D**) trials of the external familiarization task from Experiment 2. (Color figure online)

The images were 16 nonverbalizable figures (Endo et al., 2003), as used by Verschooren and colleagues (Vershooren, et al. 2019a; Verschooren et al., 2020), predefined in four sets of four figures.^3^ Each participant was randomly assigned to a predefined combination of two sets. In Experiment 2, the PsychoPy ‘noise’ function (Version 2020.1.3; Peirce et al., 2019) was used to degrade the external items (see Fig. 1D). The type of noise used was ‘White’ with standard settings except the opacity level which was fixed to 0.8.

### Procedure

The experiments were programmed in Python using PsychoPy (Version 2020.1.3) and ran online using Pavlovia (Peirce et al., 2019). Participants were instructed to take a comfortable, upright seated position in front of their computer within a normal viewing distance. Both experiments followed the same procedure except that in Experiment 2, the external items were degraded.

In this task, participants had to compare two figures and decide whether the two figures matched or not by pressing the corresponding keys. In some trials, these figures were both presented on-screen (i.e., external task). In other trials, one figure was presented on-screen and the other one was kept in memory (i.e., internal task). The study aimed to measure how efficiently participants can switch between externally observed information on-screen (i.e., external task) and internally kept information in memory (i.e., internal task). By comparing two consecutive trials of the same task (repeat trials) versus a switch between two tasks (switch trials), the switch cost can be calculated (mean RT switch trials – mean RT repeat trials). In both Experiment 1 and 2, the task consisted of two parts: a training phase and an experimental phase. The training phase contained an internal and external familiarization task and one training block of the experimental trials. After this training block, the internal and external familiarization tasks were repeated before continuing with the experimental trials. The experimental phase consisted of eight blocks of experimental trials.

Before starting the internal familiarization task, the four items had to be memorized (internalized) in their corresponding location (see Fig. 1A). More specifically, participants had 15s (indicated on the screen) to memorize the items and their corresponding locations and could choose to continue before by pressing the space bar. These four internal items were randomly assigned to each participant (see Materials section) and remained the same throughout the entire experiment for each participant. In the internal familiarization task, each trial started with a fixation cross for 350 ms. Next, participants were presented with a square consisting of four compartments, each containing a question mark (representing the previously memorized items; see Fig. 1B). One of the compartments was highlighted with a blue frame indicating the target item. Simultaneously, a probe was presented above the square, and participants had to decide whether the probe matched the highlighted target or not. The probe could correspond to the highlighted target item (i.e., valid trial) or one of the three other items (i.e., invalid trial). Participants were instructed to press ‘k’ for a match and ‘d’ for a mismatch (or vice versa) between the target and the probe. When participants made a mistake during the internal familiarization task, the internal set was represented for 5 s to allow reencoding of the items (it was never represented during the experimental trials). Thus, in the internal familiarization task, participants had to retrieve the target item (i.e., not visible on-screen), previously presented at that location, from memory and compare it to the presented probe (i.e., visible on-screen).

The external familiarization task was the same as the internal familiarization task except that here, the four items were always presented on-screen and their locations were randomized on each trial (see Fig. 1C and 1D). Thus, in the external familiarization trials, participants saw the target item on-screen (i.e., visible on-screen), and they had to compare it to the presented probe (i.e., visible on-screen). In both familiarization tasks, the trial remained on-screen for 15 s or until a response was given. Additionally, the instructions were represented after making a total of 20 mistakes to make sure the participant correctly understood the task. Afterward, the count was reset to zero. Once participants had reached an accuracy of 85% on each of these familiarization tasks and at least 18 correctly performed trials in total, participants continued with the training of the experimental trials. This criterion was chosen to make sure all participants correctly understood the task and memorized the set adequately and equally. If participants did not reach this criterion on one of the two familiarization tasks, the experiment continued after a maximum of 75 trials.^4^ After the training of the experimental trials, and before starting the first experimental block, the familiarization tasks were repeated.^5^

**Fig. 2:**
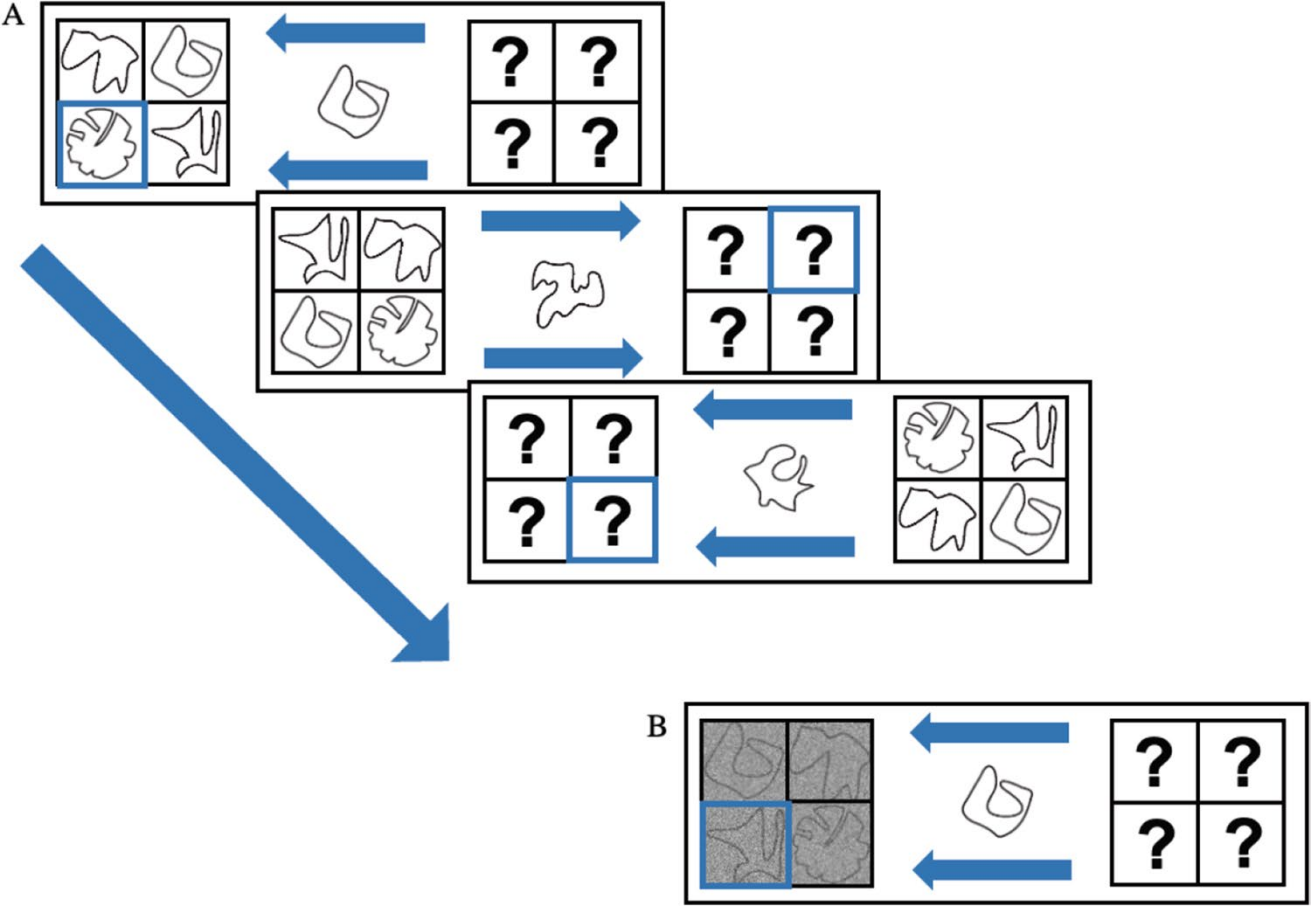
An example of (**A**) a series of trials (External, Internal, Internal) from Experiment 1, and (**B**) an External trial from Experiment 2. (Color figure online)

In the experimental task, each trial began with a fixation cross for 250 ms, followed by two squares consisting of four compartments presented on either side of the screen for 300 ms (see Fig. 2). One of these squares consisted of the external set, while the other square referred to the internal set. The position of the two sets varied from trial to trial (i.e., counterbalanced), such that, for example, the external set could be presented in the left square on one trial while it could be presented in the right square on the next trial. The square corresponding to the external set showed four figures of the external set, one in each of the compartments of the square. Their location within the square was randomized on each trial, meaning that they could be presented in any one of the four compartments on each trial. The square corresponding to the internal set consisted of four question marks, representing the four previously memorized items in the four locations. At the same time, two arrows were presented in between the squares. One above the fixation cross, and the other one below. These arrows pointed to one of the two squares—that is, the one relevant for that trial (external or internal). Additionally, one of the compartments of the relevant square was highlighted with a blue frame indicating the target item to be compared with the probe. For the external trials, the target figure was present on the screen. In the internal trials, the item was represented by a question mark on-screen instead and participants were instructed to retrieve the item previously presented at the location of the question mark from memory. After 300 ms, a central probe replaced the fixation cross for 2,500 ms (or until response) while all other stimuli remained on-screen. Participants had to match the presented probe to the highlighted target. Taken together, this resulted in four types of trials; an internal trial followed by an internal trial (i.e., internal repeat trial), an external trial followed by an external trial (i.e., external repeat trial), an external trial followed by an internal trial (i.e., internal switch), or an internal trial followed by an external trial (i.e., external switch).

The main task consisted of 8 experimental blocks. Each block started with two warm-up trials followed by 80 experimental trials. The order of the experimental trials was counterbalanced within each block to make sure there was an equal number of trials in each of the four trial types (i.e., external repeat, external switch, internal repeat, internal switch). For each of these four trial types, there was an equal amount of match and mismatch trials (i.e., probe–target compatibility). For each trial, there was a .50 chance of the external figures to be presented on the left or right side of the screen and a .25 chance for each of the four locations to be highlighted as the target item (with exclusion of immediate target or probe repetitions). The order of the eight experimental blocks was randomized while the practice block remained the same for each participant^6^. Trial sequence was predetermined in five different versions to meet the restrictions described above (i.e., trial sequences were pseudorandomly generated). Each participant was randomly assigned one of these five versions. Additionally, at the beginning of the experiment, each participant was randomly assigned a familiarization task order for the training (i.e., starting with the internal or external familiarization task), a combination of four internal and four external figures (see ‘Materials’), and a response mapping (i.e., ‘d’ as match, ‘k’ as mismatch or vice versa).

All the experimental files, raw data, and analysis files can be found on OSF (https://osf.io/hfc9z/).

## Data analysis and results

Following our preregistration, after collecting data from 60 participants per experiment, participants with less than 75% accuracy in the experimental trials were excluded.^7^ This resulted in a final sample of 42 and 53 participants^8^ in Experiments 1 and 2, respectively. In both experiments, and like Verschooren et al. (2020), we removed the training trials, warm-up trials, the trials on which an error was made, and trials preceded by an error. Analyses were preregistered beforehand and done in R using the BayesFactor package (Morey & Rouder, 2018) with default settings^9^. Detailed descriptive results of Experiments 1 and 2 can be found in Appendix 2.

**Fig. 3:**
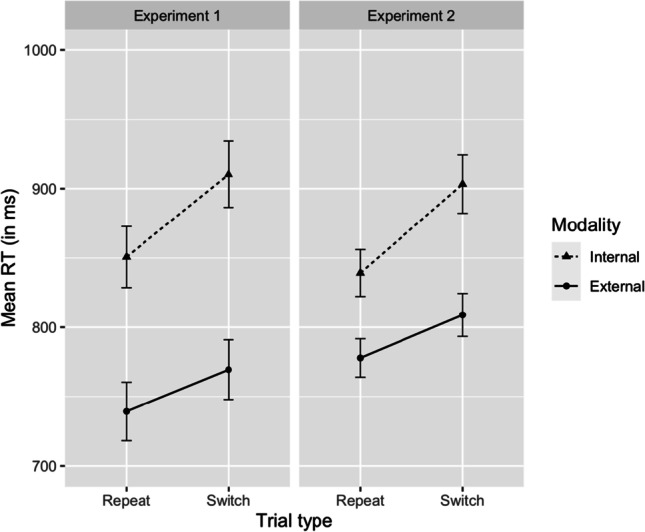
Mean RT (in ms) presented with standard error bars for the external and internal repeat and switch trials

**Fig. 4:**
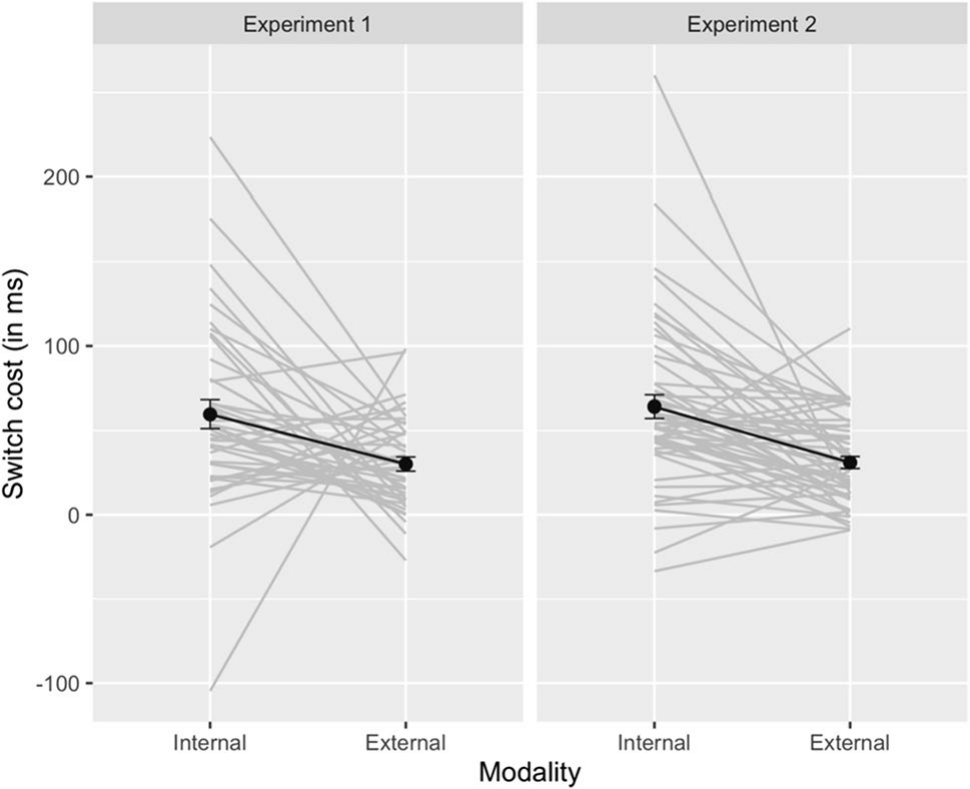
The internal and external switch cost (in ms) for each participant (grey) and the overall mean (black) presented with standard error bars

Firstly, we ran a Bayesian repeated-measures ANOVA on RTs, with modality (internal vs. external) and trial type (repeat vs. switch) as within-subjects variables. In Experiment 1, we found extreme evidence for including the main effects of modality (BF_10_ = 3.94 × 10^25^) and trial type (BF_10_ = 17951) in the best model, respectively, but evidence for the interaction remained inconclusive (BF_01_ = 1.26; see Fig. 3). Additionally, we ran a Bayesian paired one-sided *t* test to assess the evidence for the expected asymmetry in switch costs (RT difference switch trials – repeat trials, per modality). This showed strong evidence (BF_10_ = 11.19) for a larger internal than external switch cost (59 ms vs. 30 ms; see Fig. 4).

In Experiment 2, the results of the same BANOVA showed very strong evidence for including the main effects of trial type (BF_10_ = 5077×10^4^) and modality (BF_10_ = 1.67×10^18^) in the best model, but the evidence for including the interaction was inconclusive (BF_10_ = 2.41; see Fig. 3). Furthermore, we ran a Bayesian paired two-sided *t*-test to investigate whether there is a substantive difference between the internal and external switch cost. This was indeed the case (BF_10_ = 1457). We followed up with an additionnal unpreregistered Bayesian paired one-sided *t*-test, like in Experiment 1, which showed very strong evidence (BF_10_ = 2914) for a larger internal than external switch cost (64 ms vs. 31 ms; see Fig. 4).

In addition, we analyzed the data of Experiments 1 and 2 together.^10^ This revealed that the best model included the main effects of modality, trial type, and experiment as well as the interactions between modality and experiment, and between modality and trial type. The results showed very convincing evidence for including the interaction between modality and experiment in the best model (BF_10_ = 1392), demonstrating that our manipulation of stimulus strength in Experiment 2 was successful. Degraded external items were more difficult to process, resulting in decreased RTs. Additionally, model comparison showed moderate evidence in favor of including the interaction between modality and trial type in the best model (BF_10_ = 8.62), demonstrating the presence of an asymmetrical switch cost. Finally, we observed strong evidence against including the critical three-way interaction between modality, trial type, and experiment (BF_01_ = 29.81), which contrasts sharply with the predictions of the stimulus strength account. Further details of this analysis can be found in Appendix 1.

## Discussion

The current study aimed to replicate the asymmetrical switch cost as observed by Verschooren and colleagues (Verschooren et al., 2020; Verschooren, Liefooghe, et al., 2019a) in Experiment 1, and investigate whether this asymmetry could be explained by differences in the representational strength in Experiment 2. The results of Experiment 1 demonstrated an asymmetrical switch cost, although the evidence was more subtle than previously observed (Verschooren et al., 2020; Verschooren, Liefooghe, et al., 2019a). The results from Experiment 2 again showed a subtle asymmetrical switch cost and did not support the idea of differences in representational strength between internal and external items.

### Evidence for a subtle asymmetrical switch cost

Against our expectations, the BANOVA in Experiments 1 and 2 did not show evidence for the interaction between modality (internal vs. external) and trial type (repeat vs. switch). However, we did observe strong evidence for a larger internal switch cost in the one-sided *t* test in each experiment. Moreover, the results of the combined data of both experiments showed an interaction between modality and trial type. Thus, we observed convincing evidence for an asymmetrical switch cost, but only when tested for directly (using a Bayesian one-sided *t* test) or when combining the data of both experiments (95 participants). Overall, in line with the findings from Verschooren and colleagues (Verschooren et al., 2020; Verschooren, Liefooghe, et al., 2019a), we observed subtle evidence for an asymmetrical switch cost between the internal and external focus of attention. These findings are consistent with previous studies that have investigated attentional switches between memory and perception (e.g., Carlson et al., 1993; Weber et al., 1986) and demonstrated that switching from perception to memory is slower than the other way around (e.g., Dark, 1990).

### What might be causing this asymmetrical switch cost?

In Experiment 2, we tested whether decreasing the difference in stimulus strength between internal and external items results in a reduction or abolishment of the asymmetrical switch cost. This was not the case, indicating no evidence for a contribution of stimulus strength in the observed pattern. One limitation is that a between-experiment, and thus between-subjects comparison, was used to test the effect of stimulus degradation, whereas a within-subjects design could have allowed for a more direct comparison within participants. Another limitation is that we did not equate baseline performance (i.e., RTs on repeat trials). However, the asymmetrical switch cost has also been demonstrated when the mean RTs for the internal repeat trials were faster than the external repeat trials, or almost equal (Verschooren, Liefooghe, et al., 2019a). Finally, our manipulation in Experiment 2 might not have been strong enough to eliminate the asymmetrical pattern. While we acknowledge this possibility, we did observe indirect confirmation that our manipulation was successful, as the RTs for the external trials were increased in Experiment 2 compared with Experiment 1. Therefore, at least some modulation of the switch cost pattern would be expected if stimulus strength plays a role in the asymmetrical switch cost.

So, if not stimulus strength differences, what might be causing this asymmetrical switch cost? Although further disentanglement of possible accounts goes beyond the scope of the current paper, there are already some promising accounts that could explain these results. For one, as suggested and investigated by Verschooren et al. (2020), the asymmetrical switch cost might be due to associative interference. However, to be able to explain the asymmetrical switch cost by means of an associative interference account, one needs to accept that the internal task is the easier, more dominant task. This still seems counterintuitive considering that RTs were slower, and accuracy was lower (see Appendix 1) for the internal compared with the external task in Experiments 1 and 2. An alternative explanation, more closely related to the stimulus strength hypothesis, would be in terms of stimulus similarity and interference. Indeed, we believe that the similarities between internal and external items might be a source of interference, causing the observed asymmetrical switch cost. More precisely, the items used for the internal and external sets are drawn from the same pool of abstract figures (Endo et al., 2003). When attention needs to be refocused from the external to the internal set, interference might be emerging from the external items present on-screen. In the literature, several studies have already shown that a perceptually presented, plausible interference (i.e., items drawn from the same pool of the memory items) can have disruptive effects on working memory performance (e.g., Ueno, Allen, et al., 2011a; Ueno, Mate, et al., 2011b) and on an item in the internal focus of attention specifically (e.g., Allen & Ueno, 2018; Hu et al., 2014; Hu et al., 2016). Thus, it is possible that when participants need to compare the presented probe with the memory item, the memory item is (partly) interchanged with one of the external items viewed right before. Alternatively, when attention needs to switch from internal to external, we expect little to no interference from the internal items onto the external items as the latter are presented on-screen, leaving little to no room for interchangeability. To some degree, this explanation is similar to the associative interference account proposed by Verschooren et al. (2020). However, the critical difference lies in the fact that their account concerns task-level interference, whereas this alternative explanation concerns item-level interference.

Yet another explanation was provided by Dark (1990), who suggested that the increased RTs for the switch from external to internal may be due to a memory retrieval cost. More specifically, participants were slower to switch from an external to an internal item compared with the other way around, but this difference disappeared when a pre-cue was shown, indicating which modality would have to be recalled first. Thus, when participants were aware of the task order, and could potentially select the memory item beforehand, the increased switch cost disappeared. Therefore, Dark (1990) interpreted the observed pattern as a cost for item selection rather than for attentional switching between modalities. Verschooren et al. (2020) investigated a similar retrieval cost account in their Experiment 3. When the external task was replaced by an interruption task consisting of simple mathematical equations, thereby creating a task switch in the absence of a competing attentional set while memory retrieval is still required for the internal set, the asymmetrical switch cost completely disappeared. If participants were indeed slower to switch from perception to memory because of a memory retrieval cost (i.e., items need to be brought into the focus of attention), it should not matter if participants are doing a similar external task (Experiment 1, Verschooren et al., 2020) or a mathematical interruption task (Experiment 3, Verschooren et al., 2020). In both cases, one would expect a similar cost for bringing an item into the focus of attention, if any. However, when a mathematical interruption task was introduced, no asymmetrical switch cost was observed (see Verschooren et al., 2020, for more information). Thus, it seems that a memory retrieval cost cannot explain the asymmetrical switch cost, although further research is needed to refute this hypothesis with more certainty.

Taken together, our findings confirm the presence of a subtle asymmetrical switch cost between the internal and external focus of attention, for which evidence can only be convincingly gathered when tested most directly (using a Bayesian paired one-sided *t* test) or when a large amount of data is available (95 participants across two experiments). Degrading the external stimuli did not modify this pattern and thus, we observed no evidence for a contribution of differences in representational strength between internal and external stimuli in the asymmetrical switch cost. Alternatively, it could be that our manipulation was not strong enough and so, future research could aim to use different, stronger manipulations to test this hypothesis as well as attempt to disentangle alternative accounts of the subtle, asymmetrical switch cost associated with attentional switching between perception and memory.

## Figures and Tables

**Fig. 5:**
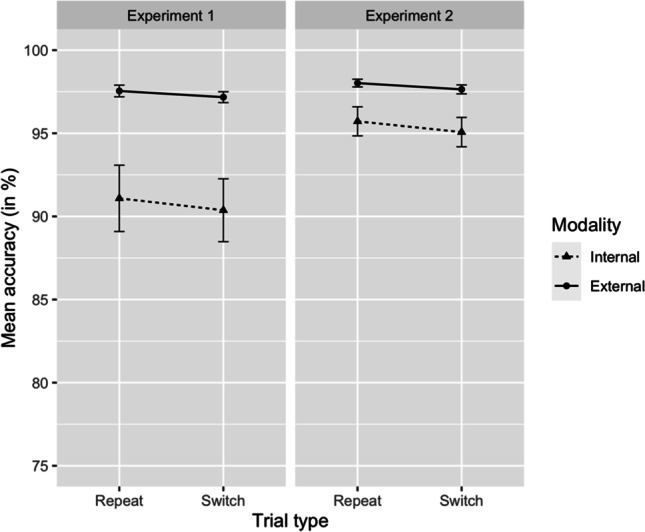
Mean accuracy for the internal and external repeat and switch trials, presented with *SE* bars

## Data Availability

The raw data, experimental files, and analysis files for Experiment 1 and 2 are available on the Open Science Framework (https://osf.io/hfc9z/). Both experiments were preregistered, see https://osf.io/75t4m for Experiment 1 and https://osf.io/ud96e for Experiment 2.

## Appendix 1

### Exploratory analysis: Accuracy

As an additional unpreregistered exploratory analysis, we ran a repeated-measures BANOVA on accuracy, with modality (external vs. internal) and trial type (repeat vs. switch) as within-subjects variable using JASP with default settings (JASP Team, 2020), for each experiment separately. These analyses were done for the participants included in the overall analyses described in the main text, i.e., after applying the preregistered exclusion criteria (except that here, we kept trials on which an error was made, and trials preceded by an error).

In Experiment 1, the best model only included the main effect of modality (BF_10_ = 514241). There was moderate evidence against the main effect of trial type (BF_01_ = 5.48) and clear evidence against the interaction (BF_01_ = 24.6). Descriptively, participants had higher accuracy scores for the external repeat trials compared with the internal repeat trials, and higher accuracy scores for the external switch trials compared with the internal switch trials (see Appendix Fig. 5).

In Experiment 2, the best model also only included the main effect of Modality (BF_10_ = 7575), and again, we found moderate evidence against the main effect of Trial type (BF_01_ = 3.91) and clear evidence against the interaction (BF_01_ = 18.14). The descriptive results were also very similar to the ones of Experiment 1, such that participants had higher accuracy scores for the external repeat trials compared with the internal repeat trials, and higher accuracy scores for the external switch trials compared with the internal switch trials (see Appendix Fig. 5).

Taken together, the results are very similar for Experiments 1 and 2. In both experiments, it seems that overall, accuracy was higher for the external trials compared with the internal trials, but there was no interaction between modality and trial type. Indeed, accuracy barely decreased from repeat to switch trials, and this was the case for both the external trials (Experiment 1: 98% for repeat and 97% for switch trials, Experiment 2: 98% for repeat and 98% for switch trials) as well as the internal trials (Experiment 1: 91% for repeat and 90% for switch trials, Experiment 2: 96% for repeat and 95% for switch trials). So even though participants were slower to respond on switch trials (see RT analysis), their memory accuracy did not change meaningfully, which suggests that there was no speed–accuracy trade-off.

### Frequentist statistics analyses

In addition to our preregistered analysis, we ran two repeated-measures ANOVAs on RTs with modality (external vs. internal) and trial type (repeat vs. switch) as within-subjects variables in each experiment, using JASP with default settings (JASP Team, 2020). In Experiment 1, the results displayed a main effect of modality, *F*(1, 41) = 94, *p* < .001, ${\eta}_p^2$ = .70, a main effect of switch, *F*(1, 41) = 113.46, *p* < .001, ${\eta}_p^2$ = .74, and an interaction effect between modality and switch, *F*(1, 41) = 8.17, *p* = .007, ${\eta}_p^2$ = .17. In Experiment 2, the results showed a main effect of modality, *F*(1, 52) = 52.13, *p* < .001, ${\eta}_p^2$ = .50, a main effect of switch, *F*(1, 52) = 122.78, *p* < .001, ${\eta}_p^2$ = .70, and an interaction effect between modality and switch, *F*(1, 52) = 23.16, *p* < .001, ${\eta}_p^2$ = .31.

### Analysis of combined data in Experiments 1 and 2

We conducted a preregistered Bayesian repeated-measures ANOVA on RTs, with modality (internal vs. external) and trial type (repeat vs. switch) as within-subjects factors and experiment (regular stimuli vs. degraded stimuli) as a between-subjects factor. In this BANOVA, we observed that the best model included the main effects of modality, trial type, and experiment, and the interactions between modality and trial type and modality and experiment. Model comparison showed very convincing evidence for including the main effect of trial type (BF_10_ = 4.03×10^13^), modality (BF_10_ = 5.77×10^47^), and experiment (BF_10_ = 640) in the best model. Next, we observed very strong evidence (BF_10_ = 1392) for including the interaction between modality and experiment in the best model. Participants were faster to respond to the external trials in Experiment 1 compared with Experiment 2 (mean difference = 44 ms), while the mean RT for the internal trials remained the same in Experiment 1 and Experiment 2 (mean difference = 1 ms). This demonstrates that our manipulation of the external items in Experiment 2 was indeed successful, such that degraded external items were more difficult to process and result in decreased RTs. Additionally, model comparison showed moderate evidence (BF_10_ = 8.62) in favor of including the interaction between modality and trial type in the best model. Participants were overall faster to respond to internal repeat trials compared with internal switch trials and faster to respond to external repeat trials compared with external switch trials. Finally, the critical three-way interaction between modality, trial type, and experiment was not present in the best model, and we observed strong evidence (BF_01_ = 29.81) against including this triple interaction (i.e., the full model) in the best model.

## Appendix 2

Table 1

**Table 1:** Mean reaction times and standard errors for Experiment 1, Experiment 2 and the Combined data

	Modality	Trial type	Mean RT (in ms)	SE (in ms)	RT cost (in ms)
Experiment 1	Internal	-	867	3	118
	External	-	749	2	
	-	Repeat	783	2	47
	-	Switch	830	3	
	Internal	Repeat	851	22	59
		Switch	910	24	
	External	Repeat	739	21	30
		Switch	769	22	
Experiment 2	Internal	-	868	2	75
	External	-	793	2	
	-	Repeat	806	2	48
	-	Switch	854	2	
	Internal	Repeat	839	17	64
		Switch	903	21	
	External	Repeat	778	14	31
		Switch	809	16	
Combined data	Internal	-	868	2	94
	External	-	774	1	
	-	Repeat	796	1	48
	-	Switch	844	2	
	Internal	Repeat	834	2	66
		Switch	900	3	
	External	Repeat	761	2	26
		Switch	787	2	

## Footnotes

1 See, for example, Gade et al. (2014) and Oberauer et al. (2013) for more information on procedural versus declarative working memory.

2 Internal items were to-be-memorized beforehand and were only presented a few times at the beginning of the experiment (see Methods for more details).

3 We divided the 16 figures into four groups of four figures (A, B, C, and D). Next, we predefined four combinations of these sets: internal set A with external set B, internal set B with external set A, internal set C with external set D, and internal set D with external set C. In this way, we randomized the use of the different figures as either internal or external items.

4 In the internal familiarization task, eight participants in Experiment 1 and seven participants in Experiment 2 went through 75 trials in the first repetition of the familiarization tasks. Of these participants, five in Experiment 1 and five in Experiment 2 were included in the final dataset after applying the preregistered exclusion criteria. In the external familiarization task, only one participant from Experiment 2 went through 75 trials in the first repetition of the familiarization tasks. This participant was included in the final dataset after applying the preregistered exclusion criteria. None of the abovementioned participants went through 75 trials of either one of the familiarization tasks in the second repetition of these tasks.

5 Average performance for the external familiarization task was 97% in Experiment 1 and 95% in Experiment 2, and for the internal familiarization task, this was 89% in Experiment 1 and 90% in Experiment 2.

6 This is different from the original study in which there were nine blocks of experimental trials and the order of the nine blocks was randomized with the first presented block being used as the practice block.

7 Exploratory, unpreregistered analyses of the accuracy scores can be found in Appendix A.

8 Following the preregistration, we analyzed the data of both experiments after the first 40 participants (26 and 35 participants in Experiment 1 and 2, respectively, after applying the preregistered data exclusion criteria). The BANOVA showed that the best model included the main effects of modality and trial type for both experiments. Since the evidence against including the critical interaction in this best model remained inconclusive in Experiment 1 (BF_01_ = 1.69) and 2 (BF_01_ = 1.47), we continued collecting data in both experiments as preregistered.

9 See Appendix A for the not-preregistered frequentist statistics version of the analysis.

10 This analysis was preregistered for Experiment 2 because our aim was to examine whether the asymmetrical pattern would vary between the original experiment (using regular external stimuli) and our second experiment (using degraded external stimuli). Moreover, combining the two experiments allowed us to test the asymmetrical switch cost pattern in a larger dataset.
